# Thermal Personalities of Older People in South Australia: A Personas-Based Approach to Develop Thermal Comfort Guidelines

**DOI:** 10.3390/ijerph17228402

**Published:** 2020-11-13

**Authors:** Helen Bennetts, Larissa Arakawa Martins, Joost van Hoof, Veronica Soebarto

**Affiliations:** 1 School of Architecture and the Built Environment, The University of Adelaide, North Terrace, Adelaide, SA 5005, Australia; larissa.arakawamartins@adelaide.edu.au (L.A.M.); veronica.soebarto@adelaide.edu.au (V.S.); 2 Faculty of Social Work & Education, The Hague University of Applied Sciences, Johanna Westerdijkplein 75, 2521 EN Den Haag, The Netherlands; j.vanhoof@hhs.nl; 3 Institute of Spatial Management, Faculty of Environmental Engineering and Geodesy, Wrocław University of Environmental and Life Sciences, ul. Grunwaldzka 55, 50-357 Wrocław, Poland

**Keywords:** housing, cluster analysis, thermal comfort, design guidelines, heating, cooling

## Abstract

An important consideration for future age-friendly cities is that older people are able to live in housing appropriate for their needs. While thermal comfort in the home is vital for the health and well-being of older people, there are currently few guidelines about how to achieve this. This study is part of a research project that aims to improve the thermal environment of housing for older Australians by investigating the thermal comfort of older people living independently in South Australia and developing thermal comfort guidelines for people ageing-in-place. This paper describes the approach fundamental for developing the guidelines, using data from the study participants’ and the concept of personas to develop a number of discrete “thermal personalities”. Hierarchical Cluster Analysis (HCA) was implemented to analyse the features of research participants, resulting in six distinct clusters. Quantitative and qualitative data from earlier stages of the project were then used to develop the thermal personalities of each cluster. The thermal personalities represent different approaches to achieving thermal comfort, taking into account a wide range of factors including personal characteristics, ideas, beliefs and knowledge, house type, and location. Basing the guidelines on thermal personalities highlights the heterogeneity of older people and the context-dependent nature of thermal comfort in the home and will make the guidelines more user-friendly and useful.

## 1. Introduction

The ability to stay in one’s community and age-in-place is the preferred strategy for most people, including the majority of older Australians [1]. For some people, this may mean moving to a smaller or more manageable dwelling as they approach older age. For others, it may mean making adjustments to their existing homes. The World Health Organization’s (WHO, Geneva, Switzerland) Checklist of Essential features of Age-friendly Cities notes that appropriate accommodation is important for the independence and quality of life of older people and that the accommodation should be “appropriately equipped to meet the ambient environmental conditions” [2] (p. 31). This could refer to the design of the house itself and the way it can respond to the environment, or to the heating and cooling equipment installed to assist with the thermal comfort of the occupants. Either approach requires an understanding of local conditions: the climate, housing, and the thermal preferences and behaviours of older people in their homes.

The majority of thermal comfort studies have been conducted with younger people and in non-residential settings such as offices or climate chambers [3]. Studies exploring whether the thermal comfort requirements of older people differ from those of younger people are inconclusive. One review of both climate chamber studies and field studies concluded that there were no significant differences between the comfort temperatures of young and older people once clothing, metabolic, and anthropometric differences were taken into account [4]. Other studies reported that older people preferred higher temperature [3,5,6], or lower [7,8], that their comfort range was narrower [9] or wider [10]. Despite differences in individual studies, both physiological changes (for example, changes to the metabolic rate and cardiovascular system) and behavioural changes (for example, decline in activity, more time spent at home) suggest older people have increased vulnerability to prolonged periods of both high and cold temperatures [11].

While there are a number of design guidelines that provide information about modifying an existing home or building a new one to suit ageing-in-place (for example, Livable Housing Australia [12]), thermal comfort is rarely referred to. The focus is mainly on improving accessibility, supporting self-care, and preventing falls and injury [13]. On the other hand, guidelines and standards for thermal comfort [14,15,16] rarely address older people or thermal comfort in the home. This is despite knowledge about the impact of ageing on people’s thermoregulation [17,18,19,20,21,22], about the connection between the thermal conditions in the home and older people’s health and well-being [23], as well as about the problems of rising energy prices and increasing energy poverty amongst older people [24]. It appears many older people themselves are not aware of these issues. In a recent survey of older people in South Australia, 85% said they had not heard, seen, or received information about how hot and cold weather could affect their health, while 90% said they had not heard, seen, or received information about how to improve their thermal comfort [25].

The research, *Improving the thermal environment of housing for older Australians* (ARC DP180102019), investigates the thermal comfort and thermal behaviours of people aged 65 and over who live independently in South Australia. Data have been collected via telephone survey of 250 respondents [25], focus group discussions with 49 participants about their heating and cooling behaviour [26], and indoor environmental monitoring and occupant surveys in 57 households involving 71 older occupants to capture their attitudes, behaviours, and approaches to achieving thermal comfort [27,28]. The previous stages of this research project revealed the inter-individual differences in the research participants as well as their housing conditions, thermal preferences, (environmental) attitudes and concerns, and heating and cooling practices. In total, 340 people, aged 65 or older who were living independently, participated in the project. The participants exhibited the diversity evident in older people in terms of demographics, with ages from 65 to over 93, and various levels of income, education, health, and well-being. Their thermal behaviours and thermal preferences varied greatly along with the location and thermal performance of their dwellings [29,30].

An important outcome of the project will be thermal comfort guidelines for older people who wish to age-in-place in South Australia. Based on the research data, the guidelines are intended to raise awareness of the links between older people’s well-being and thermal comfort in the home and to provide information about keeping cool in hot weather and warm in cold weather. Rather than focusing only on specifying a range of temperatures that are suitable for older people, the guidelines will describe a variety of strategies for achieving thermal comfort, taking into consideration people’s personal factors, housing conditions, and knowledge, as well as the actions that older people prefer to take.

Focus groups with older people undertaken earlier in the research project revealed that the strategies employed by individuals to keep cool in hot weather and warm in cold weather were complex, inter-related, and influenced by a range of issues including personal factors and preferences, people’s beliefs and experiences, the design and location of the dwelling, the type of heating and cooling equipment, as well as their financial concerns [26]. Some people discussed that their thermal behaviours were mainly influenced by the cost of heating and cooling, while others indicated that they were affected by concern over the impact of the thermal environment on their health and well-being. Recognising these different motivations will be an important aspect of the design guidelines.

**Figure 1:**
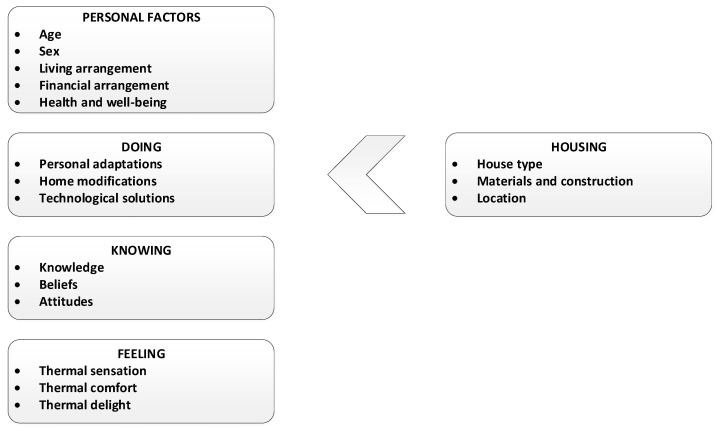
Factors important for understanding heating and cooling behaviours. Adapted from van Hoof et al. [26].

Analysis of the data derived from focus group discussions identified four key concepts influencing thermal behaviour: personal factors, doing, knowing, and feeling [26]. The four key concepts highlight the importance of aspects of the housing and lead to important domains and subdomains (Figure 1).

This paper describes the method used to develop the organisation and content of the guidelines. Based on the concept of personas, a number of discrete “thermal personalities” were developed through cluster analysis of quantitative data (for example, personal factors, doing, and housing) from the survey and the monitoring questionnaires, supplemented with qualitative information (for example, about knowing and feeling) from the interviews with the participants of the monitoring and thermal comfort survey and from the focus group discussions.

Personas are “fictitious, specific and concrete representations of target users” [31]. Originally proposed by software developer Alan Cooper [32] as a way to represent the goals and motivations of different types of prospective software users, personas are typically developed from both quantitative and qualitative data and are presented as fictional characters with a name, appropriate image, and a narrative. Personas can be used to summarise and communicate research about people [33], to link such research with design ideas [34], and can stimulate empathy or understanding of user perspectives, particularly where these perspectives are different from those of the designer’s [35].

The thermal personalities developed in this study reflect strategies that older people currently use to achieve thermal comfort in the South Australian context and highlight where additional information will be beneficial. Basing the design guidelines on a number of different thermal personalities derived from the experiences and opinions of the participants themselves highlights the different approaches that people have to thermal comfort.

## 2. Methodology

In order to develop the thermal personalities, cluster analysis was used to identify groups amongst the study participants. In cluster analysis, similar objects are grouped into clusters such that the clusters are distinct from each other, while the members within the cluster are broadly similar to each other [36]. The clustering was based on data from 250 participants of the telephone survey [25] and 71 participants of the indoor environmental monitoring/occupant survey [27,28,29,30]. Note that only a small number of the 49 participants of the focus group were included in the cluster analysis, as the rest did not participate in either the telephone survey or the monitoring, where the detailed questionnaire was administered. Further, a number of people participated in all three—the telephone survey, focus group discussions, and indoor monitoring—hence, they were only counted once. This resulted in 303 total participants for the clustering.

After carefully going through all the questions used in the telephone survey and occupant survey, a set of 18 questions were identified as relevant features with which to judge the similarity between each participant in the clustering analysis. Some adjustments on how the data were analysed are explained below.

As the data collection stages—i.e., the telephone survey and indoor environmental monitoring—covered different combinations of questionnaires, the first step of the process involved sorting and separating only the questions that were repeated in both stages and, where possible, combining questions. For example, people were asked to rate their concern about the cost of heating as well as their concern about the cost of cooling on a five-point scale from 1 = not at all concerned, 2 = somewhat concerned, 3 = concerned, 4 = very concerned, to 5 = extremely concerned. As more than 90% of people responded in the same way to both questions, the categories were combined to depict their *Concern about the cost of heating and cooling*. The scale was reduced to a three-point scale, i.e., 1 = not at all concerned, 2 = concerned (covering the previous votes 2 and 3), 3 = very concerned (corresponding to the previous votes 4 and 5). Where the original responses differed for heating and cooling, they were checked. In some cases, the respondent did not use either heating or cooling, so the response for the one they had was recorded to represent the concern over the cost of energy. Otherwise, any responses that included 4 or 5 in the old scale were recoded as 3 (very concerned) in the new scale. Other cases were recorded as the lower value.

During the survey, many respondents had chosen “Declined to answer” when asked about the annual household income, although all had provided the source of income (i.e., either working full or part-time, part or full government-funded aged pension, or self-funded retiree). Note that a self-funded retiree is someone whose retirement income is derived from a contribution-based benefit known as superannuation in Australia or from other sources such as investments or savings. For cases where household income was not given, an amount was calculated based on the source of income that was provided with amounts based on the current means-tested aged pension in Australia for either a single person (AUD 24,000/year) or couple (AUD 36,000/year). Thus, the first category for *Annual household income* (<AUD 30,000) corresponds to a person who lives alone and receives the aged pension. The middle category equates to a single person on part-pension with additional income (for instance, working part-time or investments) or for a couple receiving either the full government pension or a part-pension supplemented by other income. The category for >AUD 50,000 applies for people who do not qualify for the pension as their income from other sources (for instance, superannuation or savings) is too high. The validity of this approach was checked against the cases where both household income and the source of income had been provided.

Both the survey and monitoring questionnaire included separate questions about whether specific health symptoms had been diagnosed by a doctor in either hot or cold weather. While some symptoms were particular to either hot or cold weather (for instance, heat stroke, dehydration, pneumonia), most symptoms occurred in both hot and cold weather (for instance, asthma, bronchitis, renal or kidney condition, heart condition). Thus, these were only counted once per person but combined in the feature ‘Weather-affected health symptoms’.

The EQ-5D-5L is a health-related quality of life questionnaire about mobility (the ability to walk about), self-care (washing and dressing oneself), usual activities (work, housework, family or leisure activities), pain or discomfort, and anxiety or depression [37]. Respondents were asked to rate their problems in each category on a scale from 1 (no problems) to 5 (extreme problems or unable to do).

Both the survey and monitoring questionnaire included the question, “When it’s very hot, what is the first thing you do to cool down?”. Individual responses were grouped for the feature ‘First action to keep cool’ into personal (i.e., drink water, wear light clothes, reduce activities, stay inside, take cool showers, go for a swim); household (i.e., pull down blinds, shut curtains, keep windows and doors shut during the day or open them up when it cools down); and technology (i.e., turn on fan or air conditioning). Similarly, responses to ‘First action to keep warm’ were grouped as personal (i.e., hot drinks or hot food, wear warm clothes, keep active, stay in bed longer, use knee rugs, take hot showers); household (i.e., open curtains during day, close them at night, shut doors between rooms); and technology (i.e., turn on heater).

Table 1 shows the 18 features, their data types, and the scales or categories used. The data included different data types: ordinal (such as age group) and nominal (such as sex and living arrangement).

The clustering algorithm used was an agglomerative (bottom up) hierarchical cluster analysis (HCA) performed using Anaconda v2019.3 [38], Python v3.7, NumPy, and SciPy libraries. HCA was chosen as there was initially a large number of variables and this approach is considered appropriate for high dimensional and low sample size (HDLSS) data. [39,40]. Additionally, unlike techniques such as k-means, it does not assume or determine the number of clusters (k) in advance but rather, produces a dendrogram diagram that represents the similarity or distance between clusters.

As there were mixed data types used for the clustering analysis (i.e., nominal and ordinal data), each feature required a different distance measure to calculate the similarity between each person. The Gower dissimilarity measure [41] is able to deal with mixed type data types and, therefore, was chosen for this procedure.

The HCA started with all 303 people as separate individual clusters and progressively merged them according to a pre-determined linkage criterion. This criterion determines which distance measure to use between data points. For this study, three linkage criteria, namely weighted, average, and complete [42], were tested in parallel to each other, in order to help choose the best performing cluster merging strategy. The silhouette score [43] was calculated for the different numbers of clusters ranging from 2 to 13. The silhouette score (from −1.0 to +1.0) shows how similar an object is to its designated cluster compared to all the other clusters, with +1.0 representing the best results and negative values representing bad clustering outputs. On consideration, the results for 4–7 clusters were examined in more detail as these appeared the most appropriate number of clusters for the guidelines, while providing enough variation to highlight the difference amongst the participants. For these, Pearson’s Chi-squared (Χ^2^) tests were performed to calculate the significance of the differences between clusters considering each feature.

Once the cluster analysis was finalised, the thermal personalities were developed based on the salient features of each cluster. These were determined by examining the percentage breakdown in each category of each feature. Where the percentage was 75–99%, it was deemed that the category was “highly likely” for that cluster; between 51 and 74%, it was deemed “likely”. Where no category exceeded 50%, it was either deemed not salient for that feature or, if appropriate, categories were combined. The thermal personality narratives were further developed with quotes from the focus groups about qualitative aspects of thermal comfort. Information from the monitoring participants not included in the questionnaires (for example, about house design and construction and the way heaters and coolers are operated) was also used to enrich the narratives.

## 3. Results

### 3.1. Cluster Analysis Results

The cluster analysis identified that with six clusters, the silhouette score was greater than 0.0 (0.1), and the clusters were considered significantly different in all 18 features tested through Pearson’s Χ^2^ tests (*p* < 0.05) (Table 2).

**Figure 2:**
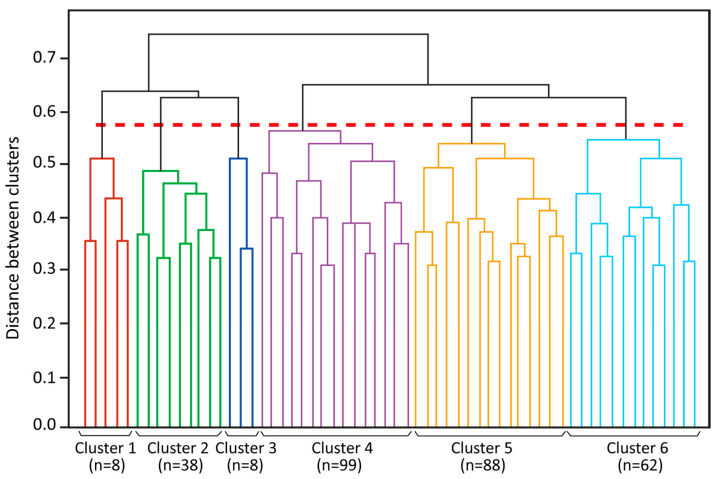
Clustering dendrogram.

Figure 2 shows the dendrogram that resulted from the clustering process, truncated to improve visualisation. The dashed horizontal line indicates the height at which the final six clusters, using all 18 features and complete linkage criteria, can be identified. The *y*-axis of this dendrogram reflects the distance between different clusters, from 0 to 1.0. The height (y) at which any two clusters are joined represents how similar they are. Two clusters that are joined at a low y-level are more similar to each other than the ones joined at higher levels.

The results of the cluster analysis are shown in the Supplementary Materials and the description of the salient characteristics of the clusters is shown in Table 3.

### 3.2. Thermal Personalities

Based on the salient characteristics of each cluster, the narratives for the thermal personalities were developed, as shown in Table 4. A name was assigned for each personality (not a real participant’s name) plus, where appropriate, other aspects of personal factors, doing, knowing, feeling, and housing were incorporated to emphasise the identity and indicate specific thermal behaviours. The transcripts of the focus group discussions and the audio recordings of the monitoring participants were examined for quotations to enrich the narratives, particularly in relation to the qualitative aspects of thermal behaviour. These appear in italics in the text of Table 4.

### 3.3. Development of Thermal Comfort Guidelines

The narratives of each cluster were then used to identify the thermal comfort guidelines that could be relevant for that cluster. The design guidelines are intended to improve people’s knowledge (i.e., knowing), particularly about home modifications and technological solutions (i.e., doing), as well as aspects of housing that can affect thermal comfort and energy use.

Table 5 presents the important points for the guidelines of each cluster. Note that the details of the actual guidelines are outside the scope of this paper and will be reported elsewhere.

## 4. Discussion

Based on the concept of personas, this study has identified thermal personalities of older people living independently in South Australia as a basis for producing thermal comfort guidelines for people ageing-in-place in this region. Personas have been used widely in software development, marketing, and product design [44]. In recent decades, the rise of person-centred policies along with an increasing computerisation of information has seen the use of personas spreading. For example, in the health sector, personas have been used when developing public health messages and eHealth platforms for patients [45,46,47], during the design of medical equipment [48,49,50], and when developing health policy [51]. Personas of older people have been incorporated in health informatics [52,53,54]. A project in Europe has developed 30 basic senior personas as a tool for software developers using data derived from the longitudinal survey SHARE (Survey of Health Ageing and Retirement in Europe) [55]. Taşoz and Afacan developed three personas of older people that were used to explore simulated ageing and the effect on basic activities of daily life [56].

In the area of building design and thermal comfort, Jais et al. [57] and McCracken et al. [58] developed personas of people living with dementia to inform architects and designers. Goldstein et al. [59] used inferred and invented personas to develop occupant behaviour models for thermal simulation, while Haines and Mitchell [60] drew on evidence collected from a 4-year study of energy-saving technologies for owner-occupiers to develop personas of different approaches to domestic energy retrofitting. A similar approach was adopted by Ortiz and Bluyssen, who developed five archetypes of residential energy users and suggested that the archetypes will be useful for refining building simulation models and also for building designers wishing to develop building features for specific energy-using archetypes [61].

For this study, the thermal personalities were developed by analysing the characteristics of 303 research participants, and their responses to a range of questions related to how they deal with the weather and how they operate their homes. These characteristics and responses are referred to as features. Using cluster analysis, participants with similar features were grouped together. Whilst the analysis resulted in six distinct clusters, there were a number of challenges in the process. First, data were imbalanced. For example, initially, the participants were grouped into five age groups (65–69, 70–74, 75–79, 80–84, and 85 and over) but upon looking into the data more closely, there were very few participants aged 65–69; thus, a decision was made to reduce the age groups to three (65–74, 75–84, 85 and over). Reducing to three age groups also provided clearer distinctions in terms of age-related thermal behaviour, housing decisions, and health and well-being.

The second challenge was about the number of features included in the cluster analysis. Initially, more than 30 features were considered for the analysis. Many were rejected as the data were incomplete or were consistently evenly distributed (for instance, preference for either hot or cold weather did not show any difference). Indeed, many of the participants had similar characteristics. For example, in line with the general Australian population, the vast majority lived in a separate house built of brick veneer. While using fewer features resulted in a higher silhouette score, indicating that the clusters were distinctive from each other, the results were not comprehensive enough to be useful as a basis for the design guidelines. The process of identifying the features involved constant trying out, considering the outcome.

After a number of iterations, using the 18 features resulted in six distinctive clusters. These features are related to thermal comfort in complex and inter-related ways. For example, thermal comfort requirements and actions are likely to be different for the different age groups. People in the age group 85 years and over are likely to have lower income, more health and well-being issues, and are more likely to live alone than those aged 65–74 [62]. Some chronic health conditions that are common in the older population can be affected by either hot or cold weather. This may cause discomfort or changes to daily routines at one level but, in extreme cases, may lead to hospitalisation or mortality during heat waves or prolonged cold [63,64,65,66].

In addition, there may be age-related changes to housing. The majority of people downsizing and moving to a retirement home in South Australia are in the age group of 65–74 [67]. Age may also affect decisions about modifications to the house. During the focus group discussions, it emerged that a person’s age was one consideration they took into account when assessing whether it was “worth” doing something, such as installing solar panels to reduce energy costs in light of “how much time is left” (to live). Similarly, the type of housing may determine what modifications are possible. Compared to an owner-occupied home, in a retirement home, the ability to make changes to the fabric of the house (such as adding external shading) is severely constrained.

Along with age, numerous studies have identified living arrangement as a risk factor for heat-related morbidity and mortality, with older people who live alone particularly vulnerable during heat waves [68,69]. In terms of thermal comfort, households with more than one person may have occupants with different thermal requirements (for example, for medical reasons) or different thermal preferences. On the other hand, a household with more than one person is likely to have financial advantages with a higher household income plus a lower energy use and cost per person.

The household income may constrain what is possible in terms of capital changes to a house, the sort of heating and cooling technology that is affordable, and the attitudes to energy usage for heating and cooling. The level of concern that people have about heating and cooling costs is likely to be a driver of thermal comfort behaviour. The costs may not be simply financial costs. A few people indicated that they were “not concerned” about the cost of heating or cooling because they felt the financial cost was less important than the potential cost to their health of being too hot or too cold, while some people were “very concerned” about the environmental cost of heating and cooling energy use rather than the financial cost.

The approach described in this paper of using different thermal personalities as the basis for the guidelines evolved in response to the previous stages of the research, where it became apparent that older people had a range of approaches to thermal comfort depending on their particular circumstances. Complex issues are at play, including the local context (climate, house type, heating and cooling equipment) and the individual’s personal situation, habits, beliefs, and preferences. One person may eschew air-conditioning for cooling because of their environmental beliefs, while another may use energy-intensive whole-house heating for many months of the year finding it alleviates their arthritis. In both cases, appropriate (but different) information may help them reduce energy costs and improve their health and well-being.

These design guidelines will differ from existing guidelines and traditional thermal comfort standards, such as ASHRAE Standard 55 [14], ISO 7730 [15], and ISO/TS 14415 [16]. Thermal comfort research has been dominated by building science and quantitative analysis, often with a view to defining or developing standards of optimal environmental conditions. For example, the WHO recommends temperatures between 18 and 24 °C to be the “safe” temperature range where “there is no demonstrable risk to human health of healthy sedentary people” [70]. However, the WHO recommends a minimum temperature higher than 18 °C for older people. On the other extreme, temperature between 25 and 32 °C is recommended by WHO as acceptable maximum temperatures [71]. While recent studies conducted both in environmental chambers [6,72,73] and in dwellings [9,74] have started to make distinctions between thermal comfort for younger and older people, most did not consider people’s behaviour in their own home and solely focussed on one’s thermal responses. Adopting both quantitative and qualitative approaches, this study, on the other hand, has considered the older people in the true context of their home. This includes their personal factors (i.e., age, sex, living arrangement, financial situation, and health and well-being status), their knowledge, belief, and attitudes, their feeling and sensation about their surroundings, their behaviours and adaptive practices, as well as the interactions with their home environment. It is expected that the guidelines that will be developed from this approach will be more useful in addressing the heterogeneity of older people.

The study, however, has a number of limitations. Firstly, people volunteered to take part in the study and this introduced a bias in terms of people who were interested in research and willing to commit for a long time-frame study. Most were in (relatively) good health. Thus, although the study participants were still heterogeneous, there were some groups who were under-represented in the study including those in poor health, renters, people from non-English speaking backgrounds, and people living in different house types.

Secondly, while the focus group discussions [26] provided qualitative information useful to enrich the understanding from the results of the initial survey [25], the questions posed to the participants during the focus group discussions were not all the same as those used in the survey due to the flow of the discussions at the time. There could have been greater standardisation of questions asked at the different stages of the research and better integration of the non-quantitative aspects.

Thirdly, as there are only three climate zones in South Australia (BSk, Csa, and Csb climate zones according to the Köppen–Geiger climate classification [75]), the results of the study are particularly relevant only to these climates. Similar research in other climate zones is likely to reveal different thermal behaviours and this, in turn, may lead to the identification of different thermal personalities. For instance, someone in northwest Europe living in a Cfb climate may not have to consider the extreme heat that sometimes occurs in South Australia but would have to deal with much lower temperatures. In other words, the thermal behaviours and thermal comfort requirements seen in the participants of this study may be quite different from those living in climates other than the BSk, Csa, and Csb climate zones.

Along with the climate, there are many aspects of thermal comfort that are particular to the local context such as housing tenure and construction methods, heating and cooling equipment, and energy costs. Nevertheless, while the focus of this study is on older people in the particular context of South Australia, the approach can be adopted in other contexts and for other groups in society. One approach could be to conduct a process that is close to the cross-cultural validation of instruments and scales [76]. Such a validation would account for cultural differences, for instance, for older people who arrived in Australia as migrants, or for older people in other countries, or when accounting for existing forms of social inequality and deprivation [77]. This applies not only to ethnic or cultural diversity, but also to other factors such as sex, age, socioeconomic position, or health status, which influence what people consider important when it comes to the age-friendliness of their living environment [78], including the thermal environment of their houses. In addition, for older people in different climate zones, a “cross-climatic validation” could also be conducted, as different climatic conditions may impact behaviours, practices, and beliefs [79]. The circumstances in which societies adapt their cultural values and practices to cold, temperate, and hot climates include the availability of money to cope with the climate [79]. Countries like Australia, which cover various climate zones, could be compared to countries which are located in just one climate zone, such as the United Kingdom, or countries which have a similar diversity in climates (such as the United States of America), and which hold similar cultural practices and share a common language. Conducting a similar study in a climatically and culturally diverse country as India, where many people also speak English, could yield different outcomes in thermal personalities.

## 5. Conclusions

The research, *Improving the thermal environment of housing for older Australians* (ARC DP180102019), investigates the thermal comfort and thermal behaviours of people aged 65 and over who wish to age-in-place. It adds to our knowledge about the variety of strategies taken by older South Australians to achieve thermal comfort. This paper describes one aspect of the method used to translate the research into information about thermal comfort that is approachable and relevant for a wide audience. The concept of personas was used to develop thermal personalities that reflect personal factors, housing conditions, and knowledge as well as the actions that older people prefer to take to keep warm in cold weather and cool in hot weather. The current study has identified six thermal personalities, which will be developed in more depth for the actual guidelines. Aspects particular to the personality (for instance, preferred strategy for keeping warm or cool, house type, and location) will be incorporated in building simulations to assess the impact on comfort, cost, or energy use of design strategies for each thermal personality. This novel approach to thermal comfort research—based on personas—in turn, will lead to personalised guidelines to achieve thermal comfort in housing for older people.

## Figures and Tables

**Table 1:** Features used for development of the thermal personalities.

Feature		Scale or Categories	Data Type
Age group		1 = 65–74 2 = 75–84 3 = 85+	Ordinal
Sex		0 = female 1 = male	Nominal
Living arrangement		0 = alone 1 = with partner/others	Nominal
Annual household income		1 = less than AUD 30,000 2 = between AUD 30,000 and AUD 50,000 3 = more than AUD 50,000	Nominal
Concern re heating and cooling cost		0 = not concerned 1 = concerned 2 = very concerned	Ordinal
EQ-5D-5L dimensions health-related well-being	Mobility	1 = no problems with walking about 2 = slight problems with walking about 3 = moderate problems with walking about 4 = severe problems with walking about 5 = unable to walk about	Ordinal
	Self-care	1 = no problems washing or dressing myself 2 = slight problems washing or dressing myself 3 = moderate problems washing or dressing myself 4 = severe problems washing or dressing myself 5 = unable to wash or dress myself	Ordinal
	Usual activities	1 = no problems doing my usual activities 2 = slight problems doing my usual activities 3 = moderate problems doing my usual activities 4 = severe problems doing my usual activities 5 = unable to doing my usual activities	Ordinal
	Pain/discomfort	1 = no pain or discomfort 2 = slight pain or discomfort 3 = moderate pain or discomfort 4 = severe pain or discomfort 5 = extreme pain or discomfort	Ordinal
	Anxiety/depression	1 = not anxious or depressed 2 = slightly anxious or depressed 3 = moderately anxious or depressed 4 = severely anxious or depressed 5 = extremely anxious or depressed	Ordinal
Number of weather-affected health symptoms		0 = none 1 = 1 2 = 2 3 = 3	Ordinal
First action to keep cool		1 = personal, such as changing clothing level 2 = household, such as close/open doors, windows, curtains, or blinds 3 = technology, such as turning on cooling or fans	Nominal
First action to keep warm		1 = personal such as changing clothing level 2 = household such as close/open doors, windows, curtains, or blinds 3 = technology such as turning on heating	Nominal
Location		1 = Iron Triangle—semi arid (Bsk) 2 = Adelaide metropolitan area—warm temperate (Csa) 3 = Fleurieu Peninsula or Adelaide Hills—mild temperate (Csb)	Nominal
Age of house (years)		1 = less than 10 years 2 = 10–20 years 3 = more than 20 years	Ordinal
In a retirement village?		0 = no 1 = yes	Nominal
Type of cooler		1 = none 2 = ducted reverse cycle (RC) * 3 = split system reverse cycle (RC) 4 = ducted evaporative cooling 5 = portable cooler	Nominal
Type of heater		1 = none 2 = ducted reverse cycle (RC) 3 = split system reverse cycle (RC) 4 = electric panel or portable heater 5 = gas heater 6 = combustion or open wood fire 7 = underfloor heating	Nominal

* RC—reverse cycle air conditioner. In some countries, this is called a heat pump.

**Table 2:** Results of Pearson’s Χ^2^ test for features used in cluster analysis.

Feature	Pearson’s Χ^2^ Significance
Age	0.000
Sex	0.000
Living Arrangement	0.000
Annual household income	0.000
Concern re heating and cooling cost	0.000
Mobility	0.001
Self-care	0.000
Usual Activities	0.000
Pain/discomfort	0.000
Anxiety/depression	0.000
Number of weather-affected health symptoms	0.046
First action to keep cool	0.000
First action to keep warm	0.000
Location	0.000
Age of house	0.014
In a retirement village?	0.000
Type of cooler	0.000
Type of heater	0.000

**Table 3:** Salient characteristics of clusters.

	Cluster 1	Cluster 2	Cluster 3	Cluster 4	Cluster 5	Cluster 6
**Age**	Highly likely ** aged 65–74	*	Highly likely aged 85+	*	*	*
**Sex**	Highly likely female	Highly likely female	Highly likely female	Likely female	Highly likely female	Highly likely male
**Live alone/with partner**	Lives alone	Highly likely lives alone	Highly likely lives alone	Highly likely lives with other	Likely lives with other	Likely lives with other
**Annual household income**	Likely > AUD 50,000	Likely < AUD 30,000	Likely < AUD 30,000	Likely > AUD 50,000	*	Likely AUD 30,000–50,000
**Mobility**	Has problems with mobility	Likely has no problems with mobility	Likely has problems with mobility	Likely has no problems with mobility.	Likely has no problems with mobility.	Likely has no problems with mobility.
**Self-care**	Highly likely has no problems with self-care	Highly likely has no problems with self-care	Highly likely has no problems with self-care	Highly likely has no problems with self-care	Highly likely has no problems with self-care	Highly likely has no problems with self-care
**Undertaking usual activities**	Highly likely has problems undertaking usual activities	Likely has no problems undertaking usual activities	Likely has problems undertaking usual activities	Likely has no problems undertaking usual activities	Likely has no problems undertaking usual activities	Likely has no problems undertaking usual activities
**Pain and discomfort**	Highly likely has moderate to severe pain or discomfort	Highly likely has some pain or discomfort	Likely has some pain or discomfort	Likely has some pain or discomfort	Likely has some pain or discomfort	Likely has some pain or discomfort
**Anxiety and depression**	Likely has some anxiety or depression	Likely has no anxiety and depression	Likely has no anxiety and depression	Highly likely has no anxiety or depression	Likely has some anxiety or depression	Highly likely has no anxiety or depression
**Weather-affected health symptoms**	Likely has 1 or more	Likely has none	Likely has 1 or more	Likely has none	Likely has none	Highly likely has none
**First thing to keep cool**	Highly likely use household strategies	*	Likely use technology	*	Likely use household strategies	Likely use technology
**First thing to keep warm**	Likely use technology	Likely use personal strategies	Likely use personal strategies	Likely use personal strategies	Likely use personal strategies	Likely use technology
**Concern about cost of heating and/or cooling**	Highly likely very concerned	Likely concerned ***	Likely not concerned	Highly likely concerned	Highly likely concerned	Likely concerned
**Location**	Likely lives in Iron Triangle or Adelaide metropolitan area.	Live in all climate zones.	Highly likely live in Adelaide metropolitan area.	Highly likely live in Adelaide metropolitan area.	Likely live in Adelaide Hills or Fleurieu Peninsula	Likely live in Adelaide Hills or Fleurieu Peninsula
**Age of house**	Highly likely house > 20 years old	Highly likely house > 20 years old	Likely house > 20 years old	Likely house > 20 years old	Likely house > 20 years old	Highly likely house > 20 years old
**Heating**	*	*	Highly likely split system reverse cycle	Highly likely ducted reverse cycle	Highly likely split system reverse cycle	Highly likely split system reverse cycle
**Cooling**	Likely has ducted evaporative cooling.	*	Highly likely has split system reverse cycle	Highly likely ducted reverse cycle	Highly likely split system reverse cycle	Likely split system reverse cycle
**In retirement village?**	Does not live in a retirement village	Highly likely does not live in a retirement village	Does live in a retirement village	Highly likely does not live in a retirement village	Highly likely does not live in a retirement village	Highly likely does not live in a retirement village

* No salience. ** Highly likely = 75–99% of participants in the cluster; Likely = 51–74%; No salience = Less than 50%. *** combines categories Concerned and Very concerned.

**Table 4:** Thermal personalities.

	**1. Tina, 66, lives alone in Whyalla and is about to renovate her existing house** *I was probably much more mobile, in fact I know I was a lot more mobile even a year ago, and I am conscious that I spend a lot of time just sitting now.*	**2. Liz has a low income, lives alone, and wants to reduce her heating and cooling costs** *Oh yes, I care about the bills, when you’re on a pension you have to.*	**3. Elsie, 86, lives alone in an independent living unit (ILU) of a retirement village and would like some ideas for improving her comfort** *I am conscious of the power bill because it is expensive and it’s scary, but I won’t freeze now, I won’t freeze with my health and age.*
**Personal factors**	Recently retired, Tina sometimes has problems walking and often has pain that restricts her activities. She feels that her health is worse in cold weather and often gets colds and flu. In summer, her main problem is that she finds it hard to sleep. She has income from her superannuation.	Liz is often worried about money. She feels she is healthy but has occasional problems with pain in cold weather. *Feeling very, very cold. My limbs aren’t as flexible; difficulty in walking; and difficulty in staying warm.*	Elsie receives a government pension. She has a number of ailments and has to use a walking frame. Her eyesight is poor and she also finds she is getting rather forgetful. Elsie has help with shopping and visits the community centre once a week.
**Doing**	In cold weather, the first thing Tina does to keep warm is turn the heater on. She has electric portable heaters, which she uses in the living room and the bedroom. For cooling, Tina has ducted evaporative cooling. She uses this mostly at night, preferring to shut the house up on a hot day and stay inside.	Although Liz has a split system reverse cycle air conditioner in the living room and a portable heater in the bedroom, she prefers to keep warm by dressing appropriately and working in the garden. Liz only uses cooling during prolonged hot spells.	Elsie has a split system air conditioner in the living room. The first thing she does when she is feeling too warm, is to turn the air conditioner on. In cold weather, she prefers to wait until late afternoon before turning the heating on. She makes sure she is dressed warmly and will often use a knee rug when sitting.
**Knowing**	Tina has lived in Whyalla for a long time and is familiar with the climate. She is very concerned that her declining health will make it harder to remain in her own home and wants to make adjustments to the house while she can.	Liz is very concerned about the cost of heating and cooling and often finds it difficult to pay her electricity bills. *You pay an electricity account but you go without something else.*	Elsie had the air conditioner installed a few years ago but no one explained how to use it. *I’m never sure that I’ve got the thing set correctly, whether I’ve got the wrong instruction book. I always have difficulty, and I don’t really know where to go for assistance.*
**Feeling**	Tina finds her mood lightens on a sunny day. She particularly likes sitting in the sun on a cold day. For Tina, this is pure thermal delight.	Liz prefers hot weather. *I used to love the cold weather and not enjoy the heat so much but my bones love the heat, they don’t enjoy the cold.*	Elsie was born in England and she thinks this is why she has never liked the heat. *I certainly get grumpier. It affects my mood and my attitude in the hot weather.*
**Housing**	Tina lives in a house that was built 30 years ago. It is cavity brick with concrete-slab-on-ground and a corrugated metal roof. Tina thinks it has insulation in the ceiling.	Liz’s 100-year-old cottage has sandstone walls and timber floors. The kitchen and living area, added 20 years ago, has brick veneer walls with concrete slab on ground.	Elsie lives in a semi-detached unit built in the 1970s with cavity brick walls, timber floors, and a tiled roof. She thinks there is insulation in the ceiling.
	**4. Sophia lives with her husband in Adelaide and is about to renovate her house** *I’d just like to be able to do something about it rather than just push a button when it’s going to get hot or cold.*	**5. Peggy and her partner are planning to downsize to a township in the Adelaide Hills and want information about sustainable heating and cooling** *If I had a wish in life it would be to live in a home that was environmentally really good, faced the sun and did everything.*	**6. Joe lives with his wife in a seaside town on the Fleurieu Peninsula and is interested in upgrading his air conditioning** *I would say that my heating and cooling is an absolute priority, I’d rather eat mince if you know what I mean and still be warm or cool.*
**Personal factors**	Sophia and her husband have income from investments and their superannuation. They are healthy and active, regularly going to the gym, travelling, and entertaining family and friends. Sophia has few weather-related illnesses beyond the occasional winter cold.	Since retiring, Peggy has increased her involvement with community and environment groups. Although generally healthy and active, she feels she is slowing down and often becomes anxious both about her future and the state of the world.	Joe is a retired engineer who keeps himself fit and healthy. He has a part-pension that supplements his income from other sources.
**Doing**	Sophia’s house has ducted reverse cycle air conditioning, but Sophia and her husband prefer not to use this as a first response to hot and cold weather. Instead, they make sure they are dressed appropriately for the weather and that they keep active, particularly in cold weather. *Go outside, do something outside. Come back inside and you’ll feel warm, work in the garden.*	Peggy has an old split system reverse cycle air conditioner in the living room but rarely uses it. She prefers to dress warmly and keep active in cold weather and to use blinds and curtains to keep the sun out in hot weather. *I very rarely put on the air conditioner because I’m a terrible greenie, and I don’t like that air that blows on me.*	Joe’s first action if he feels too warm or cold is to turn the air conditioner on. He has one split system reverse cycle air conditioner in the living room and an old window unit in the bedroom but is considering upgrading these along with installing solar panels. *As I get older I must admit... I just flick a switch and it sorts my problems.*
**Knowing**	Sophia is concerned that the cost of heating and cooling is increasing. She hopes to continue living in the family home but realises they need to make some changes. She is interested in passive design and would like to incorporate some of these principles when they renovate.	Peggy is concerned about climate change and the links between energy use and global warming. While she is very concerned about the financial cost of heating and cooling, she is also concerned about the environmental cost.	Joe is very comfortable with technology. He keeps his own records of the weather and also records his energy use. He likes the sense of control that this knowledge gives him.
**Feeling**	Sophia likes the idea of having alternatives to the air conditioner to provide comfort. *I’d just like to be able to do something about it rather than just push a button when it’s going to get hot or cold.*	Peggy feels much healthier when she is connected to natural elements such as the sun and the wind. *Utilise nature, work with nature and appreciate nature.*	Joe loves living where he does because he finds the salty air healthy and he has a wonderful view of the ocean.
**Housing**	Sophia and her husband have lived in their bungalow for more than 10 years. It has brick walls, timber floors, and a tiled roof. They are planning to upgrade their house.	Peggy and her partner have lived in their current house for many years, but they are looking to move to a smaller house in an Adelaide Hills town.	Joe’s house is elevated lightweight construction with timber floors.

**Table 5:** Important points to be addressed in the thermal comfort guidelines.

**CLUSTER 1—Emphasis: Health and Well-Being**	**CLUSTER 2—Emphasis: Cost**	**CLUSTER 3—Emphasis: Comfort and Cost (Retirement Unit)**
This cluster will benefit from guidelines consisting of information about:	This cluster will benefit from guidelines consisting of information about:	This cluster will benefit from guidelines consisting of information about:
How hot and cold weather can affect medical conditions in older people (K); The benefits of simple modifications to the house, (D) for example: ○ improving ceiling insulation; ○ adding window and door seals; Options for operable windows that are easy to operate (K); Passive design principles to be incorporated in renovation, particularly to allow sun to come into the living area (K/D/F); Energy-efficient heating and cooling systems and temperature set points that would be conducive to maintaining good health (D).	How hot and cold weather can affect medical conditions in older people (K); Low cost household strategies to reduce the running cost of heating and cooling, (D), for example: ○ Installing reversible ceiling fans; ○ Using curtains or blinds to help reduce unwanted heat gain and heat loss; ○ Reducing the volume of rooms to be heated or cooled, for instance, by shutting doors or adding partitions; Energy-efficient heating and cooling systems (D).	How hot and cold weather can affect medical conditions in older people (K); Household strategies to improve comfort and reduce the need for heating and cooling (D), for example: ○ Using curtains or blinds to help reduce unwanted heat loss and heat gain; ○ Reducing draughts by improving the sealant around the windows and doors. Personal heating and cooling devices such as electric rugs or personal fans (D); How to operate a split system reverse cycle air conditioner efficiently, including thermostat settings, and when to turn it on and off (D); Easy-to-use remote controls for the air-conditioning system (D).
**CLUSTER 4—Emphasis: Comfort and Cost (Own House)**	**CLUSTER 5—Emphasis: Cost and Environment**	**CLUSTER 6—Emphasis: Comfort and Technology**
This cluster will benefit from guidelines consisting of information about:	This cluster will benefit from guidelines consisting of information about:	This cluster will benefit from guidelines consisting of information about:
How hot and cold weather can affect medical conditions in older people (K); Passive design principles for house renovation (K). Household modifications suitable for an old house (D), for example: ○ Summer shading including using deciduous plants; ○ Improving/adding sealant around the windows and doors; ○ Replacing existing glass in windows with better performance glazing, and/or; ○ Replacing existing fixed windows with operable ones; ○ Adding roof vents to the roof space; ○ Checking the insulation on the ceiling and, if needed, replacing/adding new insulation. Alternatives to the ducted system reverse-cycle air-conditioner for heating and cooling (K/D), for example: ○ Individual/split system so they only need to run the system in the room they occupy; Solar panels: size, type, payback (K).	How hot and cold weather can affect medical conditions in older people (K); Passive design principles for building or buying a new house (K), for example: ○ Living areas that face the sun (F); ○ Walls and ceiling are well-insulated; ○ Windows can be opened easily but safely (D/F); ○ Window, door frames, all cracks and joints are well-sealed; ○ Roof eaves and other shading that will block direct sun in summer but allow it to enter the spaces in winter; ○ Large spaces can be divided into small spaces to reduce heating and cooling (D); ○ Using natural materials for building construction, as much as possible, to minimise environmental impact and provide “warm” feeling (F); Solar panels: size, type, payback (K).	How hot and cold weather can affect medical conditions in older people (K); Benefits of using energy-efficient heating and cooling systems (i.e., replacing the old window unit) (K/D); Solar photovoltaic panels, size, cost, and payback (K); Smart air-conditioning control and application (K/D); Smart metering system (K); Benefits of insulation to be installed under the elevated timber floors to reduce heat loss, thus reducing the need for heating (K).

K = Knowing; D = Doing; F = Feeling.

## Supplementary Materials

The following are available online at https://www.mdpi.com/1660-4601/17/22/8402/s1, Table S1. Results of cluster analysis.

## References

[1] Judd B., Liu E., Easthope H., Davy L., Bridge C. (2014). Downsizing Amongst Older Australians, AHURI Final Report No. 214.

[2] World Health Organization (2007). Global Age-Friendly Cities: A Guide.

[3] Rupp R.F., Vásquez N.G., Lamberts R. (2015). A review of human thermal comfort in the built environment. Energy Build..

[4] Wang Z., De Dear R., Luo M., Lin B., He Y., Ghahramani A., Zhu Y. (2018). Individual difference in thermal comfort: A literature review. Build. Environ..

[5] van Hoof J., Hensen J.L.M. (2006). Thermal comfort and older adults. Gerontechnology.

[6] Schellen L., van Marken Lichtenbelt W., Loomans M.G.L.C., Toftum J., de Wit M.H. (2010). Differences between young adults and elderly in thermal comfort, productivity, and thermal physiology in response to a moderate temperature drift and a steady-state condition. Indoor Air.

[7] Bills R. Cold comfort: Thermal sensation in people over 65 and the consequences for an ageing population. Proceedings of the 9th Windsor Conference: Making Comfort Relevant, Cumberland Lodge.

[8] Tartarini F., Cooper P., Fleming R. Thermal environment and thermal sensations of occupants of nursing homes: A field study. Proceedings of the International High-Performance Built Environment Conference—A Sustainable Built Environment Conference.

[9] Hwang R.-L., Chen C.-P. (2010). Field study on behaviors and adaptation of elderly people and their thermal comfort requirements in residential environments. Indoor Air.

[10] Yang J., Nam I., Sohn J.-R. (2016). The influence of seasonal characteristics in elderly thermal comfort in Korea. Energy Build..

[11] Gasparrini A., Guo Y., Hashizume M., Lavigne E., Zanobetti A., Schwartz J., Tobias A., Tong S., Rocklöv J., Forsberg B. (2015). Mortality risk attributable to high and low ambient temperature: A multicountry observational study. Lancet.

[12] Livable Housing Australia (2017). Livable Housing Design Guidelines.

[13] Carnemolla P., Bridge C. (2018). A scoping review of home modification interventions—Mapping the evidence base. Indoor Built Environ..

[14] ASHRAE (2017). Thermal Environmental Conditions for Human Occupancy (ANSI/ASHRAE 55-2017).

[15] ISO (2005). Ergonomics of the Thermal Environment—Analytical Determination and Interpretation of Thermal Comfort Using Calculation of the PMV and PPD Indices and Local Thermal Comfort Criteria.

[16] ISO (2005). Ergonomics of the Thermal Environment—Application of International Standards to People with Special Requirements, Technical Specification (ISO/TS 14415).

[17] Cheshire W.P. (2016). Thermoregulatory disorders and illness related to heat and cold stress. Auton. Neurosci..

[18] Blatteis C.M. (2012). Age-Dependent Changes in Temperature Regulation—A Mini Review. Gerontology.

[19] Havenith G. (2001). Temperature regulation and technology. Gerontechnology.

[20] Schlader Z.J., Coleman G.L., Sackett J.R., Sarker S., Chapman C.L., Hostler D., Johnson B.D. (2018). Behavioral thermoregulation in older adults with cardiovascular co-morbidities. Temperature.

[21] van Hoof J., Kort H.S.M., Hensen J.L.M., Duijnstee M.S.H., Rutten P.G.S. (2010). Thermal comfort and the integrated design of homes for older people with dementia. Build. Environ..

[22] van Hoof J., Schellen L., Soebarto V., Wong J.K.W., Kazak J.K. (2017). Ten questions concerning thermal comfort and ageing. Build. Environ..

[23] Miller W., Vine D., Amin Z. (2017). Energy efficiency of housing for older citizens: Does it matter?. Energy Policy.

[24] Willand N., Horne R. (2018). “They are grinding us into the ground”—The lived experience of (in)energy justice amongst low-income older households. Appl. Energy.

[25] Soebarto V., Bennetts H., Hansen A., Zuo J., Williamson T., Pisaniello D., van Hoof J., Visvanathan R. (2019). Living environment, heating-cooling behaviours and well-being: Survey of older South Australians. Build. Environ..

[26] van Hoof J., Bennetts H., Hansen A., Kazak J.K., Soebarto V. (2019). The Living Environment and Thermal Behaviours of Older South Australians: A Multi-Focus Group Study. Int. J. Environ. Res. Public Health.

[27] Soebarto V., Williamson T., Bennetts H., Arakawa Martins L., Pisaniello D., Hansen A., Visvanathan R., Carre A. Development of an integrated data acquisition system for thermal comfort studies of older people. Proceedings of the 11th Windsor Conference: Resilient Comfort.

[28] Soebarto V., Williamson T., Carre A., Martins L.A. (2019). Understanding indoor environmental conditions and occupant’s responses in houses of older people. IOP Conf. Ser. Mater. Sci. Eng..

[29] Williamson T., Soebarto V., Bennetts H., Arakawa Martins L., Pisaniello D. Thermal comfort, well-being and health of older residents in South Australia. Proceedings of the 11th Windsor Conference: Resilient Comfort.

[30] Arakawa Martins L., Williamson T.T., Bennetts H., Zuo J., Visvanathan R., Hansen A., Pisaniello D., van Hoof J., Soebarto V. Individualising thermal comfort models for older people: The effects of personal characteristics on comfort and wellbeing. Proceedings of the 11th Windsor Conference: Resilient Comfort.

[31] Adlin T., Pruitt J. (2010). The Essential Persona Lifecycle: Your Guide to Building and Using Personas.

[32] Cooper A. (2004). The Inmates are Running the Asylum: Why High Tech Products Drive us Crazy and How to Restore the Sanity.

[33] Goltz S. (2014). A Closer Look at Personas: What They Are and How They Work Part 1. Smash. Magaz..

[34] Tvedebrink T.D.O., Jelić A. (2018). Getting under the(ir) skin: Applying personas and scenarios with body-environment research for improved understanding of users’ perspective in architectural design. Pers. Stud..

[35] Van der Linden V., Dong H., Heylighen A. (2016). Building Empathy: Opportunities for Introducing Future Users’ Perspectives in Architectural Design. Proceedings of the Engineering for Society 2016: Raising Awareness for the Societal Role of Engineering.

[36] Bratchell N. (1989). Cluster analysis. Chemom. Intell. Lab. Syst..

[37] EuroQol (2019). EQ-5D-5L User Guide.

[38] Anaconda (2019). Anaconda Software Distribution. Computer Software. Anaconda.com.

[39] Steinbach M., Ertöz L., Kumar V. (2004). The Challenges of Clustering High Dimensional Data. New Directions in Statistical Physics.

[40] Holden R.J., Kulanthaivel A., Purkayastha S., Goggins K.M., Kripalani S. (2017). Know thy eHealth user: Development of biopsychosocial personas from a study of older adults with heart failure. Int. J. Med. Inform..

[41] Gower J.C. (2005). Similarity, Dissimilarity, and Distance Measure. Encyclopedia of Biostatistics.

[42] Müllner D. (2011). Modern hierarchical, agglomerative clustering algorithms. arXiv.

[43] Rousseeuw P.J. (1987). Silhouettes: A graphical aid to the interpretation and validation of cluster analysis. J. Comput. Appl. Math..

[44] Floyd I.R., Jones M.C., Twidale M.B. (2008). Resolving Incommensurable Debates: A Preliminary Identification of Persona Kinds, Attributes, and Characteristics. Artifact.

[45] Vosbergen S., Mulder-Wiggers J.M.R., Lacroix J.P., Kemps H.M.C., Kraaijenhagen R.A., Jaspers M.W.M., Peek N. (2015). Using personas to tailor educational messages to the preferences of coronary heart disease patients. J. Biomed. Inform..

[46] Huh J., Kwon B.C., Kim S.-H., Lee S., Choo J., Kim J., Choi M.-J., Yi J.S. (2016). Personas in online health communities. J. Biomed. Inform..

[47] Haldane V., Koh J.J.K., Srivastava A., Teo K.W.Q., Tan Y.G., Cheng R.X., Yap Y.C., Ong P.S., Van Dam R.M., Foo J.M. (2019). User Preferences and Persona Design for an mHealth Intervention to Support Adherence to Cardiovascular Disease Medication in Singapore: A Multi-Method Study. JMIR mHealth uHealth.

[48] Lerouge C., Ma J., Sneha S., Tolle K. (2013). User profiles and personas in the design and development of consumer health technologies. Int. J. Med. Inform..

[49] Vincent C., Blandford A. (2014). The challenges of delivering validated personas for medical equipment design. Appl. Ergon..

[50] van Hoof J., Wetzels M.H., Dooremalen A.M.C., Overdiep R.A., Nieboer M.E., Eyck A.M.E., van Gorkom P.J.L.M., Zwerts-Verhelst E.L.M., Aarts S., Vissers-Luijcks C. (2015). Exploring Innovative Solutions for Quality of Life and Care of Bed-Ridden Nursing Home Residents through Codesign Sessions. J. Aging Res..

[51] Gonzalez de Heredia A., Goodman-Deane J., Waller S., Clarkson P.J., Justel D., Iriarte I., Hernández J. Personas for policy-making and health-care design. Proceedings of the DESIGN 2018 15th International Design Conference.

[52] Zhu H., Wang H., Carroll J.M. Creating Persona Skeletons from Imbalanced Datasets—A Case Study using U.S. Older Adults’ Health Data. Proceedings of the DIS’19 Designing Interactive Systems conference.

[53] Schäfer K., Rasche P., Bröhl C., Theis S., Barton L., Brandl C., Wille M., Nitsch V., Mertens A. (2019). Survey-based personas for a target-group-specific consideration of elderly end users of information and communication systems in the German health-care sector. Int. J. Med. Inform..

[54] Wildenbos G.A., Jaspers M., Schijven M., Peute L.D. (2019). Mobile health for older adult patients: Using an aging barriers framework to classify usability problems. Int. J. Med. Inform..

[55] Wöckl B., Yildizoglu U., Buber I., Diaz B.A., Kruijff E., Tscheligi M. (2012). Basic senior personas. Proceedings of the 14th International ACM SIGACCESS Conference on Computers and Accessibility—ASSETS’ 12.

[56] Taşoz Ş.M., Afacan Y. (2020). Simulated physical ageing: A prioritized persona-based model for accessible interiors in senior housing environments. Indoor Built Environ..

[57] Jais C., Hignett S., Estupinan Z., Hogervorst E., Polak-Sopinska A., Krolikowski J. (2018). Evidence based dementia personas: Human factors design for people living with dementia. Ergonomics for People with Disabilities.

[58] McCracken I., de la Harpeand R., Ruvo M.D. (2019). Developing dementia personas for user centered architectural design considerations in non-specialized contexts. Proceedings of the Dementia Lab Conference.

[59] Goldstein R., Tessier A., Khan A. Customizing the behavior of interacting occupants using personas. Proceedings of the SimBuild 2010: Fourth National Conference of IBPSA-USA.

[60] Haines V., Mitchell V. (2014). A persona-based approach to domestic energy retrofit. Build. Res. Inf..

[61] Ortiz M., Bluyssen P.M. (2019). Developing home occupant archetypes: First results of mixed-methods study to understand occupant comfort behaviours and energy use in homes. Build. Environ..

[62] McDougall K., Barrie H. (2017). South Australian Retirement Village Survey 2016.

[63] Hansen A., Bi P., Ryan P., Nitschke M., Pisaniello D., Tucker G. (2008). The effect of heat waves on hospital admissions for renal disease in a temperate city of Australia. Int. J. Epidemiol..

[64] Stewart S., Keates A.K., Redfern A., McMurray J.J.V. (2017). Seasonal variations in cardiovascular disease. Nat. Rev. Cardiol..

[65] Timmermans E.J., van der Pas S., Dennison E.M., Maggi S., Peter R., Castell M.V., Pedersen N.L., Denkinger M.D., Edwards M.H., Limongi F. (2016). The Influence of Weather Conditions on Outdoor Physical Activity among Older People With and Without Osteoarthritis in 6 European Countries. J. Phys. Act. Health.

[66] Zhang Y., Nitschke M., Bi P. (2013). Risk factors for direct heat-related hospitalization during the 2009 Adelaide heatwave: A case crossover study. Sci. Total. Environ..

[67] AIHW (2017). Older Australia at a Glance.

[68] Vandentorren S., Bretin P., Zeghnoun A., Mandereau-Bruno L., Croisier A., Cochet C., Ribéron J., Siberan I., Declercq B., Ledrans M. (2006). Heat-related mortality—August 2003 Heat Wave in France: Risk Factors for Death of Elderly People Living at Home. Eur. J. Public Health.

[69] Vaidyanathan A., Malilay J., Schramm P., Saha S. (2020). Heat-Related Deaths—United States, 2004–2018. MMWR. Morb. Mortal. Wkly. Rep..

[70] World Health Organization (1987). Health Impact of Low Indoor Temperatures: Report from a WHO Meeting, Copenhagen, 11–14 November 1985.

[71] World Health Organization (2018). Housing and Health Guidelines.

[72] Tsuzuki K., Ohfuku T. Thermal sensation and thermoregulation in elderly compared to young people in Japanese winter season. Proceedings of the 9th International Conference on Indoor Air Quality and Climate.

[73] Soebarto V., Zhang H., Schiavon S. (2019). A thermal comfort environmental chamber study of older and younger people. Build. Environ..

[74] Wang Z., Yu H., Jiao Y., Wei Q., Chu X. (2018). A field study of thermal sensation and neutrality in free-running aged-care homes in Shanghai. Energy Build..

[75] Beck H.E., Zimmermann N.E., McVicar T.R., Vergopolan N., Berg A., Wood E.F. (2018). Present and future Köppen-Geiger climate classification maps at 1-km resolution. Sci. Data.

[76] Sousa V.D., Rojjanasrirat W. (2010). Translation, adaptation and validation of instruments or scales for use in cross-cultural health care research: A clear and user-friendly guideline. J. Eval. Clin. Pract..

[77] Buffel T., Phillipson C., Rémillard-Boilard S., Gu D., Dupre M.E. (2019). Age-friendly cities and communities: New directions for research and policy. Encyclopedia of Gerontology and Population Aging.

[78] Dikken J., van den Hoven R.F.M., van Staalduinen W.H., Hulsebosch-Janssen L.M.T., van Hoof J. (2020). How Older People Experience the Age-Friendliness of Their City: Development of the Age-Friendly Cities and Communities Questionnaire. Int. J. Environ. Res. Public Health.

[79] van de Vliert E. (2007). Climates Create Cultures. Soc. Pers. Psychol. Compass.

