# Supplementary material for: Thermal Personalities of Older People in South Australia: A Personas-Based Approach to Develop Thermal Comfort Guidelines
Source: Int J Environ Res Public Health. 2020 Nov 13;17(22):8402. doi: 10.3390/ijerph17228402 (PMC7698095; doi:10.3390/ijerph17228402)
Supplement: Supplementary file 1 [file ijerph-17-08402-s001.pdf]

International Journal of
*Environmental Research and Public Health*

MDPI

# Supplementary Materials: Thermal Personalities of Older People in South Australia: A Personas-Based Approach to Develop Thermal Comfort Guidelines

**Helen Bennetts [1,*], Larissa Arakawa Martins [1], Joost van Hoof [2,3] and Veronica Soebarto [1]**

1 School of Architecture and the Built Environment, The University of Adelaide, North Terrace, Adelaide, SA 5005, Australia; larissa.arakawamartins@adelaide.edu.au(L.A.M.); veronica.soebarto@adelaide.edu.au(V.S.)
2 Faculty of Social Work & Education, The Hague University of Applied Sciences, Johanna Westerdijkplein 75, 2521 EN Den Haag, The Netherlands; j.vanhoof@hhs.nl
3 Institute of Spatial Management, Faculty of Environmental Engineering and Geodesy, Wrocław University of Environmental and Life Sciences, ul. Grunwaldzka 55, 50-357 Wrocław, Poland
* Correspondence: helen.bennetts@adelaide.edu.au; Tel.: +61-8-313-5836

**Table S1.** Results of cluster analysis.

| Clusters | 1 (n=8) | 2 (n=38) | 3 (n=8) | 4 (n=99) | 5 (n=88) | 6 (n=62) | All (n=303) |
|---|---|---|---|---|---|---|---|
| **Age** (*p* = 0.000*) | | | | | | | |
| 65-74 | 75% | 16% | 0% | 45% | 40% | 34% | 37% |
| 75-84 | 25% | 45% | 13% | 35% | 48% | 45% | 41% |
| 85+ | 0% | 39% | 88% | 19% | 13% | 21% | 21% |
| **Sex** (*p* = 0.000) | | | | | | | |
| Female | 88% | 79% | 75% | 61% | 85% | 23% | 63% |
| Male | 13% | 21% | 25% | 39% | 15% | 77% | 37% |
| **Living Arrangement** (*p* = 0.000) | | | | | | | |
| With partner/other | 0% | 3% | 13% | 78% | 68% | 66% | 59% |
| Alone | 100% | 97% | 88% | 22% | 32% | 34% | 41% |
| **Household income** (*p* = 0.000) | | | | | | | |
| <AUD 30,000 | 38% | 74% | 63% | 10% | 22% | 16% | 25% |
| AUD 30,000 – AUD 50,000 | 13% | 18% | 38% | 38% | 43% | 50% | 39% |
| >AUD 50,000 | 50% | 8% | 0% | 52% | 35% | 34% | 36% |
| **Mobility** (walking about) (*p* = 0.001) | | | | | | | |
| No problem | 0% | 55% | 38% | 62% | 51% | 53% | 54% |
| Slight problem | 38% | 29% | 25% | 24% | 27% | 19% | 25% |
| Moderate problem | 25% | 16% | 25% | 11% | 16% | 24% | 17% |
| Severe problem | 25% | 0% | 13% | 3% | 6% | 2% | 4% |
| Unable to walk about | 13% | 0% | 0% | 0% | 0% | 2% | 1% |
| **Self-care** (wash and dress self) (*p* = 0.000) | | | | | | | |
| No problem | 88% | 95% | 88% | 93% | 93% | 95% | 93% |
| Slight problem | 0% | 3% | 0% | 7% | 7% | 3% | 5% |
| Moderate problem | 0% | 3% | 0% | 0% | 0% | 2% | 1% |
| Severe problem | 0% | 0% | 13% | 0% | 0% | 0% | 0% |
| Unable to wash or dress self | 13% | 0% | 0% | 0% | 0% | 0% | 0% |
| **Usual activities** (*p* = 0.000) | | | | | | | |
| No problem | 13% | 74% | 38% | 74% | 70% | 74% | 70% |
| Slight problem | 25% | 21% | 13% | 17% | 20% | 13% | 18% |
| Moderate problem | 50% | 5% | 38% | 8% | 8% | 10% | 10% |
| Severe problem | 0% | 0% | 13% | 1% | 1% | 3% | 2% |
| Unable to undertake usual activities | 13% | 0% | 0% | 0% | 0% | 0% | 0% |

| | | | | | | | |
|---|---|---|---|---|---|---|---|
| **Pain/discomfort** ($p = 0.000$) | | | | | | | |
| No problem | 25% | 26% | 38% | 32% | 32% | 42% | 33% |
| Slight problem | 0% | 50% | 38% | 47% | 34% | 34% | 40% |
| Moderate problem | 38% | 18% | 0% | 17% | 30% | 24% | 22% |
| Severe problem | 38% | 3% | 25% | 2% | 2% | 0% | 3% |
| Extreme problems | 0% | 3% | 0% | 1% | 2% | 0% | 1% |
| **Anxiety/depression** ($p = 0.000$) | | | | | | | |
| No problem | 38% | 71% | 50% | 77% | 60% | 79% | 70% |
| Slight problem | 0% | 18% | 13% | 16% | 31% | 18% | 20% |
| Moderate problem | 38% | 11% | 38% | 5% | 8% | 3% | 8% |
| Severe problem | 13% | 0% | 0% | 2% | 1% | 0% | 1% |
| Extreme problems | 13% | 0% | 0% | 0% | 0% | 0% | 0% |
| **Weather-related medical symptoms** ($p = 0.046$) | | | | | | | |
| 0 | 38% | 66% | 25% | 72% | 59% | 76% | 66% |
| 1 | 38% | 26% | 25% | 19% | 25% | 16% | 22% |
| 2 | 13% | 8% | 38% | 8% | 10% | 5% | 9% |
| 3 | 13% | 0% | 13% | 1% | 6% | 3% | 3% |
| **First thing to keep cool** ($p = 0.000$) | | | | | | | |
| Personal strategies | 0% | 47% | 38% | 33% | 20% | 18% | 27% |
| Household strategies | 75% | 24% | 0% | 34% | 56% | 29% | 38% |
| Technology | 25% | 29% | 63% | 32% | 24% | 53% | 34% |
| **First thing to keep warm** ($p = 0.000$) | | | | | | | |
| Personal strategies | 25% | 68% | 63% | 62% | 66% | 26% | 55% |
| Household strategies | 25% | 3% | 13% | 13% | 13% | 3% | 10% |
| Technology | 50% | 29% | 25% | 25% | 22% | 71% | 35% |
| **Concern about heating/cooling cost** ($p = 0.000$) | | | | | | | |
| Not at all concerned | 13% | 47% | 63% | 15% | 23% | 42% | 28% |
| Concerned | 13% | 42% | 25% | 54% | 33% | 37% | 41% |
| Very or extremely concerned | 75% | 11% | 13% | 31% | 44% | 21% | 31% |
| **Location** ($p = 0.000$) | | | | | | | |
| Iron Triangle - semi arid (Bsk) | 50% | 21% | 13% | 3% | 16% | 23% | 15% |
| Adelaide - warm temperate (Csa) | 50% | 45% | 75% | 58% | 18% | 26% | 38% |
| Fleurieu Peninsula or Adelaide Hills - mild temperate (Csb) | 0% | 34% | 13% | 39% | 66% | 52% | 47% |
| **Age of house** ($p = 0.014$) | | | | | | | |
| <10 years | 13% | 5% | 38% | 10% | 11% | 2% | 9% |
| 11 to 20 years | 0% | 11% | 13% | 26% | 27% | 23% | 23% |
| >20 years | 88% | 84% | 50% | 64% | 61% | 76% | 68% |
| **Heating** ($p = 0.000$) | | | | | | | |
| None | 38% | 0% | 0% | 0% | 0% | 0% | 1% |
| Ducted RC** | 0% | 8% | 25% | 87% | 3% | 2% | 31% |
| Split system RC | 25% | 32% | 75% | 2% | 90% | 71% | 48% |
| Electric portable or panel | 25% | 13% | 0% | 2% | 2% | 6% | 5% |
| Gas | 13% | 29% | 0% | 7% | 0% | 16% | 10% |
| Wood fire | 0% | 18% | 0% | 0% | 3% | 5% | 4% |
| Underfloor | 0% | 0% | 0% | 2% | 1% | 0% | 1% |
| **Cooling** ($p = 0.000$) | | | | | | | |
| None | 0% | 26% | 0% | 0% | 0% | 5% | 4% |
| Ducted RC | 13% | 5% | 25% | 89% | 6% | 10% | 34% |
| Split system RC | 13% | 39% | 75% | 3% | 93% | 76% | 51% |
| Ducted evaporative | 50% | 13% | 0% | 8% | 0% | 6% | 7% |
| Window or portable | 25% | 16% | 0% | 0% | 1% | 3% | 4% |
| **In retirement village?** ($p = 0.000$) | | | | | | | |
| No | 100% | 97% | 0% | 94% | 76% | 94% | 87% |
| Yes | 0% | 3% | 100% | 6% | 24% | 6% | 13% |

* Threshold of Pearson's Chi-Square ($X^2$) test of significance considered as $p < 0.05$

** RC —reverse cycle air conditioner. In some countries, this is called a heat pump.
